# SGLT2 Inhibitors and Their Mode of Action in Heart Failure—Has the Mystery Been Unravelled?

**DOI:** 10.1007/s11897-021-00529-8

**Published:** 2021-09-15

**Authors:** Steffen Pabel, Nazha Hamdani, Mark Luedde, Samuel Sossalla

**Affiliations:** 1 Department of Internal Medicine II, University Medical Centre Regensburg, Regensburg, Germany; 2 Department of Molecular and Experimental Cardiology and Department of Cardiology, St. Josef-Hospital, Ruhr University Bochum, Bochum, Germany; 3 Department of Cardiology and Angiology, University Hospital Schleswig-Holstein, Campus Kiel, Kiel, Germany; 4 Clinic for Cardiology and Pneumology, Georg-August University Göttingen, and DZHK (German Centre for Cardiovascular Research), partner site Göttingen, Göttingen, Germany

**Keywords:** SGLT2 inhibitors, Heart failure, HFrEF, HFpEF

## Abstract

**Purpose of review:**

SGLT2 inhibitors (SGLT2i) are new drugs for patients with heart failure (HF) irrespective of diabetes. However, the mechanisms of SGLT2i in HF remain elusive. This article discusses the current clinical evidence for using SGLT2i in different types of heart failure and provides an overview about the possible underlying mechanisms.

**Recent findings:**

Clinical and basic data strongly support and extend the use of SGLT2i in HF. Improvement of conventional secondary risk factors is unlikely to explain the prognostic benefits of these drugs in HF. However, different multidirectional mechanisms of SGLT2i could improve HF status including volume regulation, cardiorenal mechanisms, metabolic effects, improved cardiac remodelling, direct effects on cardiac contractility and ion-homeostasis, reduction of inflammation and oxidative stress as well as an impact on autophagy and adipokines.

**Summary:**

Further translational studies are needed to determine the mechanisms of SGLT2i in HF. However, basic and clinical evidence encourage the use of SGLT2i in HFrEF and possibly HFpEF.

## Introduction: Clinical Background

### SGLT2 Inhibitors and Cardiovascular Outcome Trials

Sodium-glucose-cotransporter 2 inhibitors (SGLT2i) increase the urine glucose excretion by inhibiting SGLT2 in the proximal tubule of the kidney and thereby lower blood glucose levels. Initially, SGLT2i were evaluated for their cardiovascular safety in type 2 diabetes mellitus (T2DM) patients with either established atherosclerotic cardiovascular disease or with multiple cardiovascular risk factors. SGLT2i showed unexpected beneficial effects on the rate of major adverse cardiovascular events (MACE) consisting of cardiovascular events, cardiovascular death and all-cause mortality, with benefit only seen in patients with atherosclerotic cardiovascular disease and not in those without [1–5].

Interestingly, the reduction of cardiovascular events was primarily driven by a prevention of heart failure (HF) hospitalisation (reduction of ~30%) rather than atherothrombotic events [5]. Moreover, the favourable effects on mortality and HF hospitalisation became apparent already after 2–3 months of treatment, which make an improvement of atherosclerotic risk factors e.g. via glucose excretion rather unlikely to account for these effects [6].

Consequently, additional studies displayed that the beneficial effects of SGLT2i are less likely to be explained by refinement of classical secondary cardiovascular risk factors as blood pressure [7], cholesterol [8] or HbA1c levels [9–11]. Moreover, SGLT2i have been shown to improve cardiovascular outcomes in diabetic patients independent of cardiovascular risk/established cardiovascular disease [1, 5, 9], HF risk factors (i.e. assessed by the Health ABC HF Risk score) [10], diagnosed HF at baseline [12] or renal function [1, 13]. Therefore, the favourable effects of SGLT2i in these trials seem to be mediated by other mechanisms and raised the question about a particular benefit in HF patients.

### SGLT2 Inhibitors in Patients with Heart Failure

The DAPA-HF trial was the first one studying the efficacy of dapagliflozin in patients who suffer from HF with reduced ejection fraction (HFrEF, ejection fraction (EF) ≤40%, NYHA II–IV) with and without T2DM with respect to cardiovascular death or worsening HF [14]. Dapagliflozin therapy was established in addition to guideline-directed HF therapy. In 4744 HF patients, dapagliflozin reduced the primary composite outcome of worsening HF (hospitalisation or urgent intravenous therapy for HF) and death from cardiovascular causes by 26% as well as each component of the primary endpoint alone. Moreover, dapagliflozin caused an improved all-cause mortality and reduced HF symptoms (assessed by the Kansas City Cardiomyopathy Questionnaire, KCCQ) [14]. Likewise, in the EMPEROR-reduced trial enrolling 3730 patients with HFrEF (EF≤40%, NYHA II–IV) with and without T2DM, empagliflozin reduced the primary composite outcome of death from cardiovascular cause and hospitalisation for HF [15]. Most importantly, the beneficial effects on all mentioned end-points of dapagliflozin and empagliflozin in HF patients were achieved in patients with and without T2DM [14–17]. Of note, in both trials, SGLT2i also significantly reduced adverse renal outcomes [14–17]. Finally, the SOLOIST-WHF trial investigated the therapy with the combined SGLT2 and SGLT1 inhibitor sotagliflozin in 1222 patients with T2DM hospitalised for HF. Sotagliflozin significantly reduced the primary endpoint of cardiovascular death, HF hospitalisation and urgent visit for HF [18]. Focusing on all of these large randomised controlled trials, only DAPA-HF demonstrated a significantly improved all-cause mortality as a secondary outcome in HF patients. However, a recent meta-analysis combining DAPA-HF and EMPEROR-Reduced clearly indicated a consistent reduction of all-cause mortality in respective HF patients [19].

Further evidence of the favourable impact of SGLT2i in HF is given by the DEFINE-HF trial, which showed improvement in HF symptoms (based on the KCCQ) over 12 weeks in 263 patients with HF (EF≤40%, NYHA II–III) treated with dapagliflozin, irrespective of T2DM [20]. Of note, NT-proBNP was not changed after 12 weeks of treatment [20].

The effects of empagliflozin on 6-min walk tests were further tested in 312 patients with HFrEF in the EMPERIAL-Reduced trial which, however, showed no significant improvement after treatment with empagliflozin [21]. As discussed recently, based on the robust data of the EMPEROR-Reduced trial, different issues could possibly underlie the lack of efficacy in this study including small sample size and the use of the 6-min walk test as primary endpoint [22]. Interestingly, the favourable effects of SGLT2i on HF in the DAPA-HF and EMPEROR-Reduced trials were consistent concerning different subgroups with respect to sex, T2DM, renal function and existing HF therapy including angiotensin receptor-neprilysin inhibitors [14, 23, 24].

Therefore, SGLT2i have been emerged as a new therapy for patients with HFrEF with or without T2DM mellitus. Accordingly, dapagliflozin has recently been approved for the treatment of patients with HFrEF independent of T2DM. Clinical HF guidelines will very likely suggest SGLT2i as basal treatment for patients with HFrEF, as there is a theoretical data basis for a class IA recommendation with two clinical trials. However, the underlying mechanisms of SGLT2i in HF are still a matter of debate. Therefore, in this article, we discuss the evidence and the significance of the mechanism of action of SGLT2i with a special focus on their myocardial effects in healthy and diseased hearts.

## Mechanisms of SGLT2 Inhibitors in HF

### Translational Aspects for Mechanistic Investigation in HF: Back to Bench

**Fig. 1:**
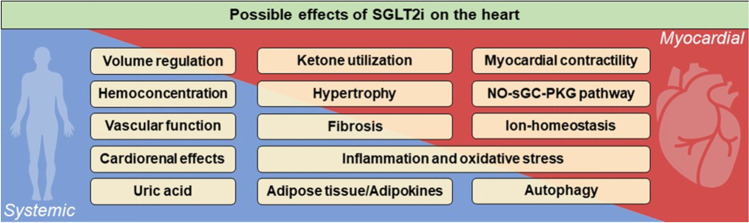
Possible mechanisms of SGLT2 inhibitors (SGLT2i) on the heart with respect to rather systemic (left panel), combined (middle panel) or myocardial effects (right panel). Image heart: © Shutterstock/Vasif Maharov; Image human: © Shutterstock/10topvector

Various mechanisms of SGLT2i have been proposed in different cardiovascular diseases over the last years. Based on clinical evidence with early beneficial effects of SGLT2i, an improvement of conventional secondary risk factors including improved glycaemic control, reduced blood pressure, weight loss or improved cholesterol seems to be unlike to explain the prognostic benefit of SGLT2i in HF [7–11, 16]. Therefore, we specifically review the potential cardiac mechanisms of SGLT2i which potentially play a favourable role in HF pathophysiology contributing thereby to improved clinical outcomes (Fig. 1).

Of note, it is important to distinguish between systemic and myocardial effects when investigating the possible mechanisms of SGLT2i in HF, as direct myocardial mechanisms cannot really be elucidated in a systemic setting where secondary factors (i.e. altered blood pressure) influence the cardiac function. Therefore, in vitro isolated hearts and myocardial tissue/cells with well-defined conditions have the advantages to study the possible direct cardiac effects of SGLT2i. Nevertheless, in vivo studies using different disease models are of translational importance to study the effects of SGLT2i in the complex, multidirectional systemic setting. It is noteworthy that the expression of SGLT2 in the heart is negligible [25, 26], while SGLT1 is expressed in the myocardium [25]. Only sotagliflozin has been shown to have a relevant inhibitory effect on SGLT1, while other SGLT2i are high selective for SGLT2 [27–29]. Nevertheless, direct cardiac effects on the heart have been demonstrated, which suggest SGLT2 independent actions in the heart.

### Volume Regulation, Haemoconcentration and Preload

Blocking SGLT2, which is strongly expressed in the proximal tubule of the kidney, causes glucosuria, natriuresis and osmotic diuresis. Empagliflozin has been demonstrated to cause a reduction in blood and plasma volume after 14 days in a cohort of 20 patients with T2DM and HF [30]. In a randomised controlled trial of 75 diabetic patients, dapagliflozin was associated with reduced blood pressure and plasma volume [31]. Canagliflozin was also shown to transiently diminish plasma volume in the first weeks of treatment [32]. However, after 12 weeks, this effect was largely attenuated [32]. Therefore, a diuretic effect of SGLT2i with consecutive favourable effects on blood volume has been proposed. A study on the diuretic effects of SGLT2i utilised a mathematical model based on data from healthy volunteers, which were treated for 7 days with dapagliflozin or bumetanide. The authors proposed a stronger reduction in electrolyte-free water and thus in interstitial fluid after dapagliflozin compared to bumetanide, which rather reduced intravascular volume [33]. The diuretic effects of SGLT2i have been proposed to regulate plasma volume without an activation of the sympathetic nervous system or the renin-angiotensin-aldosterone axis as it is known for loop diuretics [30]. This differential modulation of reducing interstitial fluid without largely affecting intravascular volume was described as the fluid hypothesis [34]. However, profound evidence is required to fully support this hypothesis.

Potentially driven by a diuretic effect of SGLT2i, systolic blood pressure and body weight were reduced after treatment with SGLT2i [31]. In line with a possible reduced plasma volume, SGLT2i have been shown to increase the haematocrit of individuals with and without T2DM [31, 32], while also having effects on erythropoietin levels (see below) [35, 36]. In reference to the diuretic effects, it has been proposed that SGLT2i reduce the preload which itself leads to improved cardiac loading conditions [34]. In line with that, cardiac magnetic resonance imaging (MRI) data showed reduced end diastolic volume in patients with T2DM after treatment with empagliflozin [37]. The reduction of interstitial fluid would beneficially affect congestion and pulmonary oedema in HF and may improve cardiac function via lowered preload.

However, the impact on plasma volume has only been reported at early time points [30–32], and the diuretic effects of SGLT2i have been demonstrated to decrease after weeks [38]. Therefore, relevant diuretic effects of SGLT2i for reducing cardiovascular mortality and HF outcomes are still questionable. Recently, a secondary analysis of the EMPEROR-Reduced trial demonstrated no differences between the effects of empagliflozin on cardiovascular mortality and HF hospitalisation in patients with (clinically assessed 4 weeks before randomisation) volume overload compared with euvolemic patients [39]. Also, markers of plasma volume regulation like haematocrit, body mass index, NT-proBNP and albumin provided no clear support for relevant diuretic properties [39]. Another argument against a prominent diuretic effect is the fact that the dose of the diuretic medication in the DAPA-HF trial could not be lowered as a consequence of dapagliflozin treatment [14, 40]. Therefore, further validation of the proposed effects of SGLT2i on volume regulation and its contribution to the cardiovascular outcomes is warranted.

### Vascular Function and Afterload

Another hypothesis by which SGLT2i could improve cardiac function is a reduction in afterload [34]. In HF, endothelial and vascular dysfunctions are present [41]. Optimising endothelial function and thus vascular stiffness might improve hemodynamic and cardiac function via lowering afterload. Interestingly, SGLT2i were suggested to improve vascular function. In a post-hoc analysis of patients with T2DM and hypertension, empagliflozin reduced blood pressure and improved markers of arterial stiffness and vascular resistance [42]. Accordingly, a small study investigating 16 T2DM patients suggested that dapagliflozin acutely improved endothelial function and vascular stiffness independent of blood pressure alterations [43]. A mechanistic study in aortic preparations from animals additionally showed that dapagliflozin exerts vasodilatory effects via protein kinase G (PKG) and voltage-gated K channels [44].

SGLT2i caused modest antihypertensive effects in different trials [7]. Effects of SGLT2i on volume regulation, arterial stiffness, natriuresis and weight loss could underlie the lowered blood pressure. However, as blood pressure would expect to improve cardiovascular risk in the long term, it appears unlikely that the modest reduction in blood pressure by SGLT2i significantly contributes to the observed early clinical outcomes [45]. Moreover, one would expect a reduction in atherothrombotic events via reduced blood pressure, but the risk for stroke was not influenced by SGLT2i [46]. Conclusively, a meta-analysis in patients with T2DM receiving SGLT2i showed no relationship between blood pressure reduction and cardiovascular events [7]. Therefore, effects of SGLT2i on blood pressure are unlikely to explain the favourable outcomes of the drugs in HF patients. Potential differential effects of SGLT2i on vascular function i.e. with respect to pulmonary circulation require further investigation.

### Cardiorenal Effects

While this review focuses on the impact of SGLT2i in HF, some potential HF-relevant mechanisms on the cardiorenal axis should be discussed as they clearly determine mortality in HF. The tremendously important prognostic significance of chronic kidney disease for cardiovascular endpoints and HF has been addressed for years and is well demonstrated [47]. A causal therapy for cardiorenal syndrome that can help improving the prognosis of patients with chronic kidney disease and cardiovascular disease beyond standard therapy with ACE inhibitors and angiotensin receptor blockers has long been lacking. SGLT2i could fill an important gap here. In this context, the DAPA CKD trial can be seen as a real milestone [48]. Patients with stage 2–4 chronic kidney diseases were included. Sixty-seven patients of the patients were diabetic, and nearly all of them were on ACE inhibitors or angiotensin receptor blockers [49]. In these patients, it was shown that taking SGLT2i not only significantly improved the prognosis of important renal parameters with respect to renal endpoints (eGFR decline above 50%, end-stage chronic kidney disease or death from renal causes), but also reduced the combined risk of death from cardiovascular causes or hospitalisation for HF by 29% in these patients. Finally, there was even a 31% reduction in all-cause mortality. These data not only point the way to an apparently highly effective therapy for chronic kidney disease, but also refer once again to the close connection between possible common pathophysiological mechanisms of cardiac and renal failure. Possible cardiorenal effects of SGLT2i will therefore be presented here.

As mentioned before, effects of glucosuria via glycaemic control and weight loss are unlikely to contribute to clinical outcomes in HF patients without T2DM. However, besides glucosuria, SGLT2i cause a natriuretic effect in the kidney. Via increased natriuresis, SGLT2 have shown to increase the amount of Na^+^ detected by the juxtaglomerular apparatus at the distal renal tubules, which causes a vasoconstriction of the afferent arteriolar vessel. As under hyperglycaemic conditions, Na^+^ transportation to the juxtaglomerular apparatus is reduced via SGLT2, the tone of the afferent arteriolar vessel is inadequately regulated [50]. Therefore, SGLT2i restore the impaired tubuloglomerular feedback mechanism [50]. Consecutively, the reduced blood flow stimulates the release of erythropoietin [51].

SGLT2i further possibly increase erythropoietin levels via hypoxia-inducible factors and utilisation of ketone bodies [51]. The SGLT2i-mediated increase in erythropoietin leads to enhanced red blood cell mass and haematocrit [31, 52], potentially associated with a reduction of renal stress and also sympathetic hyperactivity [53]. In an analysis of potential explanations of the reduced cardiovascular death in the EMPA-REG OUTCOME trial, the authors proposed that changes in haematocrit and haemoglobin, possibly related to change in volume status, mediate the diminished risk for cardiovascular death [54]. Based on the increased haematocrit, oxygen supply of the heart may be improved [51], which could theoretically increase the function of the failing heart. Of note, different renal effects of SGLT2i have been comprehensively reviewed elsewhere [55].

### Uric Acid

Uric acid is a product of the degradation of purine nucleotides and has been shown to be associated with cardiovascular diseases [56, 57]. Interestingly, uric acid is associated with elevated levels of inflammation and oxidative stress as well as reduced NO bioavailability and thus endothelial dysfunction [58]. Moreover, the renin-angiotensin-aldosterone system has been shown to be activated by uric acid [58]. SGLT2i proved a relatively robust evidence to reduce uric acid in patients with T2DM as indicated by a meta-analysis of 62 randomised control trials of SGLT2i [59]. While the effect persisted during long-term treatment, patients with chronic kidney disease (eGFR <60 mL/min) however showed no decline in serum uric acid [59]. SGLT2i reduce serum uric acid via increasing urine glucose levels which are reported to suppress urate reabsorption via SLC2A9 [56]. Although further causal mechanistic and clinical data are needed, lowering uric acid via SGLT2i might have potential cardiac benefits.

### Metabolic Effects and the Fuel Hypothesis

Since glycaemic control has no significant influence on the prognostic effects of SGLT2i, at least in the DAPA-HF and the EMPEROR-Reduced trials, it is unlikely that improvement of T2DM and hyperglycaemia-related metabolic abnormalities significantly contribute to the positive HF outcomes. However, different metabolic effects of SGLT2i have been proposed, which itself might influence cardiac function in a T2DM-independent manner.

As glucose availability is reduced upon SGLT2i, lipolysis and ketogenesis have shown to be increased in animals and patients with T2DM [60, 61]. After treatment with dapagliflozin, a reduction of visceral adipose tissue and subcutaneous adipose tissue has been shown in patients with T2DM and left ventricular (LV) hypertrophy [62]. Moreover, a shift from carbohydrate usage to lipid usage could be demonstrated in patients with T2DM [61] accompanied by an increase in circulating ketone levels such as β-hydroxybutyrate [63, 64].

As ketone bodies produce more ATP per consumed oxygen atom compared to glucose or free fatty acids, energy efficacy of the heart is higher when ketone bodies are utilised [65, 66]. Therefore, it has been postulated that the increased ketone body utilisation upon SGLT2i treatment could fuel the metabolic state of the heart and thus improve energetics [66]. Interestingly, in end-stage human HF patients, ketone utilisation is also enhanced, independent of T2DM [67]. In an interesting small, randomised trial, application of the ketone body 3-hydroxybutyrate caused an increase in EF in patients with HF without lowering myocardial external energy efficiency [68]. Moreover, metabolic regulation of branched chain amino acids (BCAAs), which is altered in HF [69], has also been reported to be affected by empagliflozin in an untargeted metabolomics approach in 25 patients with T2DM and cardiovascular disease [70]. In a porcine non-diabetic ischemic HF model (LAD occlusion), empagliflozin increased the utilisation of BCAA, free fatty acids and ketone bodies, which was associated with a mitigation of HF remodelling and improved LV function [71]. Therefore, ‘fuelling’ the heart via enhanced energy substrates upon SGLT2i might improve cardiac performance. However, the full effects of SGLT2i on ketone body utilisation, its interference with other potentially adverse pathways and finally its relevance for cardiac function and cardiovascular benefits are still controversial [72–74]. In addition to that, it has also recently been hypothesised that SGLT2i rather induce an energy-saving ‘dormancy’ programme than ‘super-fuelling’ the heart [75].

### Ventricular Remodelling and Fibrosis

Different reports suggest that SGLT2i may be involved in LV remodelling. The placebo-controlled, randomized EMPA-HEART trial studying 97 patients with T2DM, coronary artery disease, and preserved EF reported a reduction in LV mass index as obtained with cardiac MRI after 6 months of treatment [76]. Interestingly, in additional exploratory analyses, the changes in LV mass were not associated with blood pressure alterations after 6 months [76]. Likewise, empagliflozin has been demonstrated to reduce LV mass and to improve diastolic function in echocardiographic data concerning a small uncontrolled trial in T2DM patients [77]. Moreover, dapagliflozin significantly reduced LV mass in patients with T2DM [62]. In a retrospective study of diabetic patients with and without HF, SGLT2i caused an improved echocardiographic LV end-diastolic-diameter [78]. On top of that, in HFrEF patients, improved LV EF and diastolic function were suggested [78]. However, in a small randomised, double blind, placebo-controlled study of 56 diabetic patients with a previously reported reduced EF, dapagliflozin had no effects on LV end-systolic volume, LV end-diastolic volume or LV mass index obtained by cardiac MRI after 1 year [79]. Yet, the authors discussed that the study could have been limited by a small sample size and less severe HF in the patients [79]. A possible explanation is given by experimental data, in which dapagliflozin mitigated cardiac hypertrophy, apoptosis as well as fibrosis and improved LV EF in mice with pressure-induced HF [80].

Taken together, compelling evidence indicates that SGLT2i favourably affect LV remodelling in T2DM patients and in HF patients, which could be a central mechanism of the beneficial cardiac effects of SGLT2i. However, further data on potential differential effects in HFrEF and HFpEF and on the underlying mechanisms are needed.

Fibrosis plays a central role in the context of structural HF-remodelling. Interestingly, dapagliflozin exerted antifibrotic properties in a rat model of myocardial infarction by reducing reactive nitrogen and oxygen species, thereby regulating myofibroblast and M2 macrophages infiltration [81]. SGLT2i also reduced cardiac fibrosis in experimental animal models of hypertension [82] and T2DM [83]. Conversely, in 35 patients with T2DM, 6 months of treatment with empagliflozin did not change fibrosis indices as obtained by cardiac MRI. However, LV function was normal in these patients and, hence, could not be influenced positively by empagliflozin [84]. LV remodelling represents a final common pathway of different cardiac diseases. While a SGLT2i-induced mitigation of pathologic remodelling in HF shed light onto important effects in cardiovascular disease, the underlying mechanisms still need to be elucidated.

### Myocardial Contraction and Relaxation

Improving myocardial relaxation has often been proposed to be a potential prognostic mechanism of SGLT2i. Indeed, in vivo studies described effects of SGLT2i on diastolic function. In a clinical study in patients with T2DM and established cardiovascular disease (discussed above), empagliflozin improved diastolic function assessed via echocardiography [77]. Likewise, canagliflozin improved echocardiographic diastolic function in 38 patients with T2DM in a prospective observational study after 3 months of treatment [85]. Moreover, in a prospective trial in patients with T2DM and stable HF irrespective of EF, dapagliflozin was proposed to cause an improvement in echocardiographic diastolic parameters after 6 months of treatment [86] as well as in patients with T2DM [87]. MRI studies showed decreased LV end-diastolic volumes upon empagliflozin treatment in HFrEF patients with and without T2DM, while the effects on systolic parameters are controversial [37, 88, 89].

In addition to that, preclinical data support the findings of an improved diastolic function in vivo. In obese diabetic rats, which are characterised by a prolonged isovolumetric relaxation time (IVRT), acute treatment with empagliflozin mitigated diastolic dysfunction without showing effects on the LV EF [90]. In a HFpEF mouse model, empagliflozin has been demonstrated to improve diastolic function without altering systolic force, and it was associated with improved hemodynamics [91]. Moreover, in chronically treated T2DM mice models, empagliflozin beneficially affected diastolic parameters as measured by echocardiography [92, 93] or pressure catheter [94]. Conversely, another animal study reported a preserved systolic function in mice with pressure-overload-induced HF after treatment with empagliflozin, without showing effects on diastolic function [95].

However, the question about the potential mechanisms underlying the improved diastolic function upon SGLT2i arises. As discussed above, some studies reported that the improvement in diastolic function was associated with hemodynamic, metabolic or structural changes. However, these findings were not consistent in the different studies. A recent study in diabetic patients indeed showed that empagliflozin improved echocardiographic parameters of diastolic function without affecting hemodynamic parameters [96]. In vitro data (which exclude systemic confounder like plasma volume regulation or blood pressure) of direct SGLT2i-related effects on contractility are of translational importance to further clarify the underlying (cardiac) mechanisms.

**Fig. 2:**
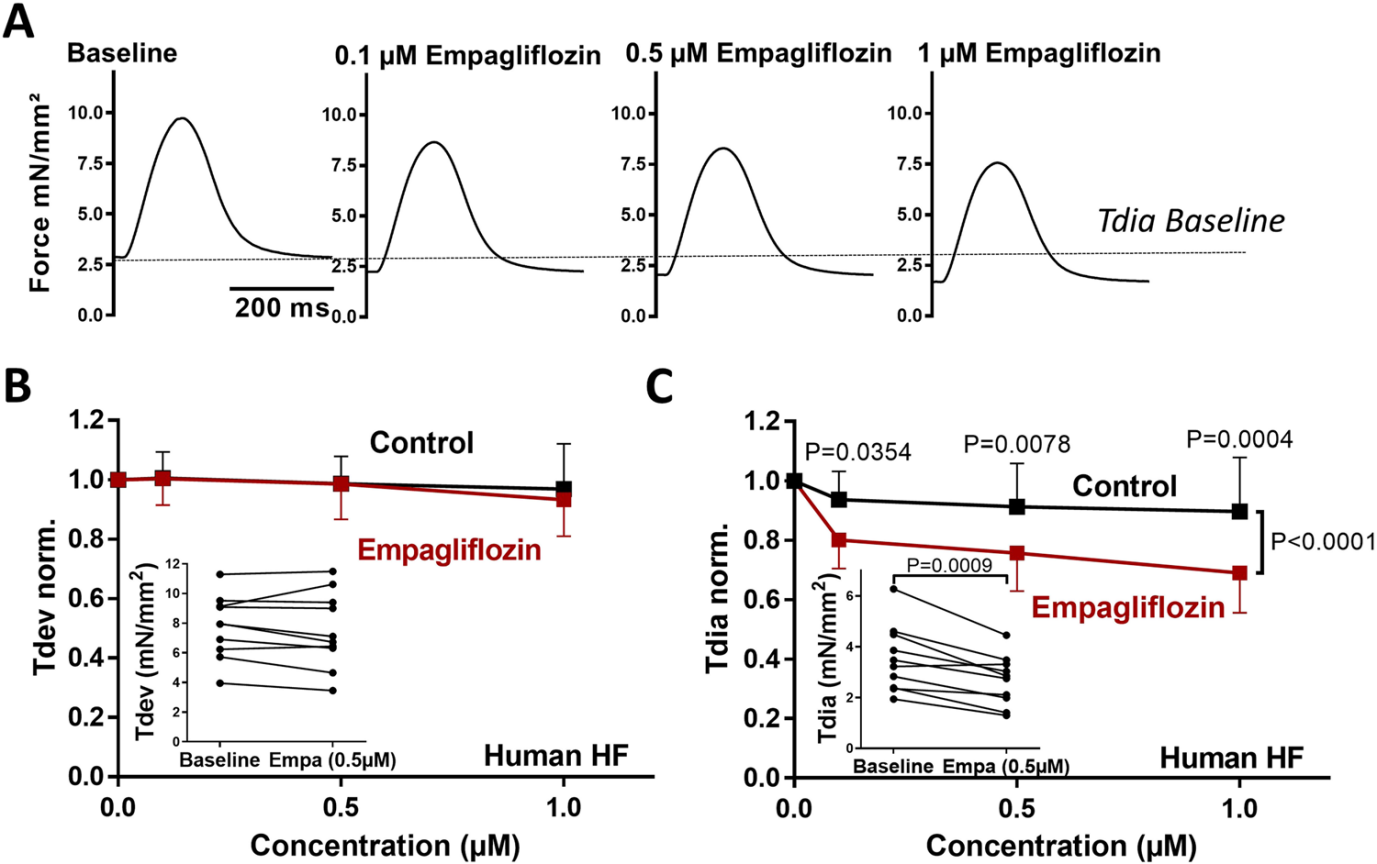
Contractility of isolatedhuman HFrEF trabecula upon empagliflozin treatment. **A** Original twitches of stimulated human trabecula before and after wash-in of increasing concentrations of empagliflozin. **B** Normalised developed (systolic) force (Tdev) and **C** normalised diastolic tension (Tdia). Raw data before and after wash-in of empagliflozin are provided in the inlay scatter. With permission from Pabel et al. [90]

**Fig. 3:**
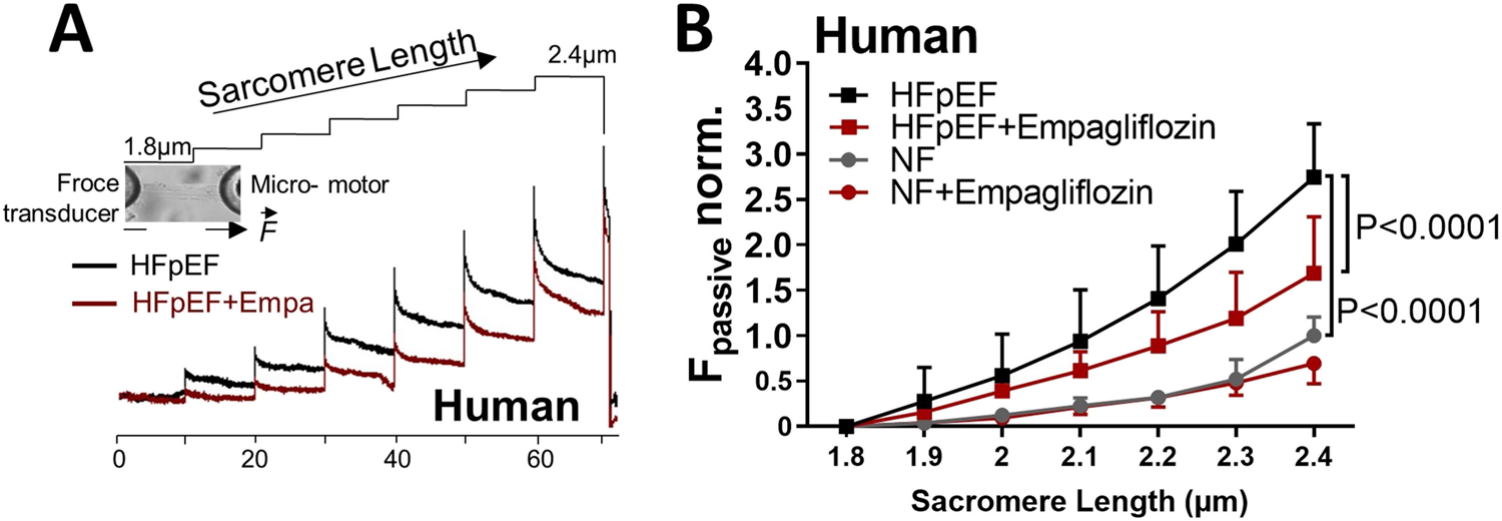
Effects of empagliflozin on the passive stiffness of (skinned) cardiomyocytes from HFpEF patients and from healthy donors. **A** The original recordings of force response during stepwise cell stretches. **B** Normalised passive stiffness of HFpEF and non-failing (NF) cardiomyocytes upon empagliflozin measured at different sarcomere lengths. With permission from Pabel et al. [90]

Our groups provided first evidence of SGLT2i-mediated effects on myocardial contractility in human ventricular trabecula from patients with HFrEF, irrespective of T2DM [90]. Empagliflozin caused a significant acute reduction of the pathologically enhanced diastolic tension in twitching human HFrEF trabecula without altering the systolic contractile force (Fig. 2) [90]. We have elucidated the underlying mechanisms of an improved diastolic function by demonstrating that empagliflozin directly reduces passive myofilament stiffness in human HFpEF myocardium (Fig. 3). These effects were mediated by an enhanced phosphorylation, typically dephosphorylated in HFpEF, of titin and other myofilament regulatory proteins via improvement of the nitric oxide (NO)-soluble guanylyl cyclase (sGC)-cGMP-dependent protein kinase (PKG) signalling pathway by empagliflozin [90, 97]. These changes were associated with altered oxidative and inflammatory pathways in human and rodent HFpEF myocardium as discussed below [97]. Also, in studies of diabetic mice, empagliflozin beneficially impacted cardiac function via NO-sGC-PKG pathway after 8 weeks of treatment [98]. As altered NO-sGC-PKG signalling is a key mechanism of diastolic dysfunction and HFpEF [99, 100], further investigations of this pathway upon SGLT2i in HFpEF appear promising [101]. Moreover, the reduced fibrotic content upon SGLT2i or changes in cardiomyocyte ion-homeostasis (see below) could also favourably affect HFpEF patients.

#### What to Expect from SGLT2 Inhibitors in HFpEF

As dapagliflozin and empagliflozin have shown to be effective for the prognostic and symptomatic treatment of HFrEF-patients independent of T2DM [102], the question about potential benefits in HFpEF arises. Current therapeutic options for HFpEF patients are limited, and no drug has yet been proven to exert prognostic relevant effects in HFpEF patients. Interestingly, a meta-analysis based on limited data of the DECLARE-TMI 58 trial and the VERTIS CV study suggests effects of SGLT2 on HF hospitalisation in patients with HFpEF [19]. In general, it can be assumed that a not insignificant number of HFpEF patients were included in the studies due to the inclusion criteria and the patient risk profile. Moreover, preclinical data on the effects of SGLT2i on diastolic function and pathways involved in HFpEF pathophysiology might also provide a rationale for the efficacy of these drugs in this complex disease [101]. The DELIVER trial (NCT03619213) investigates the effects of dapagliflozin in patients with HFpEF (EF >40%, structural heart disease, elevated NT-pro BNP levels, NYHA II–IV) on cardiovascular death or HF events. Accordingly, the EMPEROR-Preserved trial (NCT03057951) studies the effect of empagliflozin in patients with HFpEF (EF >40%, NYHA II–IV, elevated NT-proBNP, structural heart disease or HF hospitalisation) on cardiovascular death or hospitalisation due to HF. Both clinical trials will be of utmost importance, since HFpEF is still an unmet need in cardiology.

### Cardiac Ion-Homeostasis

Cardiomyocyte Ca^2+^ and Na^+^ handling fundamentally regulates excitation-contraction (EC) coupling and thus cardiac contractility [103]. Dysregulation of Ca^2+^ and Na^2+^ homeostasis is typically present in HF and contributes to contractile dysfunction and arrhythmias [104, 105]. Interestingly, direct cardiac effects of SGLT2i on cardiomyocyte ion-homeostasis have been reported. In healthy rabbit and rat cardiomyocytes, empagliflozin has been reported to acutely reduce cytosolic Ca^2+^ and Na^+^ by inhibiting the Na^+^/H^+^ exchanger 1 (NHE1) in an elevated glucose environment [106]. Interestingly, NHE1 expression is increased in human HF myocardium [107]. The inhibitory effect of SGLT2i on NHE1 was also demonstrated in human atrial tissue [108] and murine myocardium [108, 109]. Disturbed cellular Ca^2+^ and Na^+^ homeostasis in HF consisting of reduced systolic Ca^2+^ transients and elevated diastolic Ca^2+^ and Na^+^ levels in the cytosol adversely affects systolic and diastolic contractile function [110–112], impairs mitochondrial function [113], triggers proarrhythmic activity and induces electrophysiological signalling changes fuelling detrimental remodelling [105]. Therefore, by reducing cytosolic Na^+^ (and consecutively cytosolic Ca^2+^), NHE1 inhibition could favourably affect cardiomyocyte function.

Influences of SGLT2i on cardiomyocyte Na^+^ homeostasis have also recently been proposed in a study showing inhibitory effects of SGLT2i on the late Na^+^ current in murine HF cardiomyocytes [114]. The late Na^+^ current constitutes a persistent Na^+^ influx throughout the action potential and contributes to adverse electric remodelling in HF [110, 115]. Molecular docking simulations indicated that empagliflozin binds at the major cardiac Na^+^ channel isoform Na_V_1.5 and thereby reduces late Na^+^ current with little effects on peak Na^+^ current [114]. A reduction in late Na^+^ current may diminish reverse mode NCX-mediated Ca^2+^ overload and may thus reduce proarrhythmic triggers and contractile dysfunction [110]. Therefore, the inhibition of this mechanism could be a promising target in HF.

Another way how empagliflozin might counteract disturbed Ca^2+^ handling in HF has been proposed in failing ventricular murine and human cardiomyocytes. Empagliflozin treatment for 24 h reduced the activity of Ca^2+^/calmodulin-dependent protein kinase IIδ (CaMKII) [116], which is centrally involved in adverse myocardial remodelling in cardiac disease and contributes to arrhythmias [117–119]. In this study, reduction of CaMKII mitigated diastolic Ca^2+^ leak from the sarcoplasmic reticulum [116], which contributes to contractile dysfunction and arrhythmias [118]. While the reduction of CaMKII activity could be a secondary effect of empagliflozin in the (cultured) cells, this mechanism may nevertheless inhibit the vicious circle of SR Ca^2+^ leak-dependent CaMKII activation and may thereby soothe adverse electric remodelling in HF.

Yet, other studies on the impact of SGLT2i on cardiomyocyte Ca^2+^ homeostasis reported contrasting evidence which may be explained by different experimental protocols such as time of drug treatment and type of cardiomyocytes. Acute exposure with empagliflozin did not alter systolic Ca^2+^ transient or diastolic cytosolic Ca^2+^ in isolated human HF cardiomyocyte [90]. In a blinded study using healthy human-induced pluripotent stem cell cardiomyocytes long-term treatment for 2 months did also not affect Ca^2+^ homeostasis and EC-coupling proteins [120]. Of note, this is the only study on ion homeostasis with a treatment duration according to the time course of the clinical effects (~2 months). With respect to NHE1 inhibition, a recent study demonstrated that acute exposure with empagliflozin had no effects on the NHE1 function or the Na^+^ homeostasis in healthy isolated rat cardiomyocytes [121]. Differences in species and treatment protocols might limit the comparability of these studies. Thus, it is important to consider the disease/species of question when studying the effects of SGLT2i on ion-homeostasis. Finally, as Ca^2+^ and Na^+^ handling is closely intertwined with many other signalling cascades (i.e. oxidative stress, mitochondrial function), also secondary effects of SGLT2i on ion-homeostasis are conceivable.

### Oxidative Stress and Inflammation

Inflammation and oxidative stress play a central role in HF development and progression. Both are associated with increasingly prevalent comorbidities such as chronic kidney disease or metabolic syndrome [122]. Particularly in HFpEF, inflammation and oxidative stress were shown to cause structural and functional diastolic dysfunction [122, 123]. Interestingly, human HFpEF myocardium treated with empagliflozin in vitro exhibited reduced markers of oxidative stress (i.e. H_2_O_2_, GSH, LPO) and inflammation (ICAM, VCAM, TNFα and IL-6) [97].

Indeed, in several studies, SGLT2i showed anti-inflammatory and anti-oxidative properties [124] as dapagliflozin reduced the inflammasome and fibrosis in mouse models of T2DM [125] and myocardial infarction [81], ipragliflozin mitigated biomarkers of oxidative stress and inflammation in diabetic mice [126, 127] and empagliflozin attenuated oxidative stress (associated with metabolic changes) in mice after myocardial infarction [128]. As increased inflammation and oxidative stress have been shown to diminish myofilament phosphorylation and therefore worsen diastolic function, improvement of inflammation and oxidative stress may serve as an explanation for the enhanced NO-sGC-PKG pathway after SGLT2i leading to a mitigated diastolic stiffness of the LV in HFpEF. In line with that, empagliflozin improved cardiac function in diabetic mice via improvement of PKG, oxidative stress and apoptosis [98]. Moreover, in a co-culture of cardiac microvascular endothelial cell with cardiomyocytes, empagliflozin reversed TNFα-mediated microvascular dysfunction leading to enhanced NO availability [129].

### Autophagy and Adipokines

As recently discussed, a potential explanation for the reduction of inflammation and oxidative stress could be the effects of SGLT2i on autophagy [73]. Experimental data demonstrated restored autophagy in different tissue/cells, which could mitigate oxidative stress and inflammation [130, 131]. The improvement in autophagy has been postulated to be mediated by adenosine monophosphate-activated protein kinase (AMPK), sirtuin‐1 and hypoxia‐inducible factors [132]. Furthermore, it was reported that differences concerning AMPK activation are also associated with anti-inflammatory properties of SGLT2i in diabetic mice [125]. However, further mechanistic evidence for the effects of SGLT2i on autophagy, also with respect to cardiac function, is needed.

Another possible explanation for the favourable impact of SGLT2i on inflammation and oxidative stress could be an influence on the adipokine profile and leptin [133]. Adipokines are secreted i.e. from the epicardial adipose tissue and have been shown to contribute to obesity/metabolic syndrome-related remodelling and cardiovascular disease [134–136]. SGLT2i have been demonstrated to reduce epicardial adipose tissue and leptin, which was associated with reduced inflammation and oxidative stress [137, 138].

Finally, beneficial effects of erythropoietin on oxidative stress and inflammation, in particular in the metabolic syndrome, are possible [51, 139].

## Conclusion

**Fig. 4:**
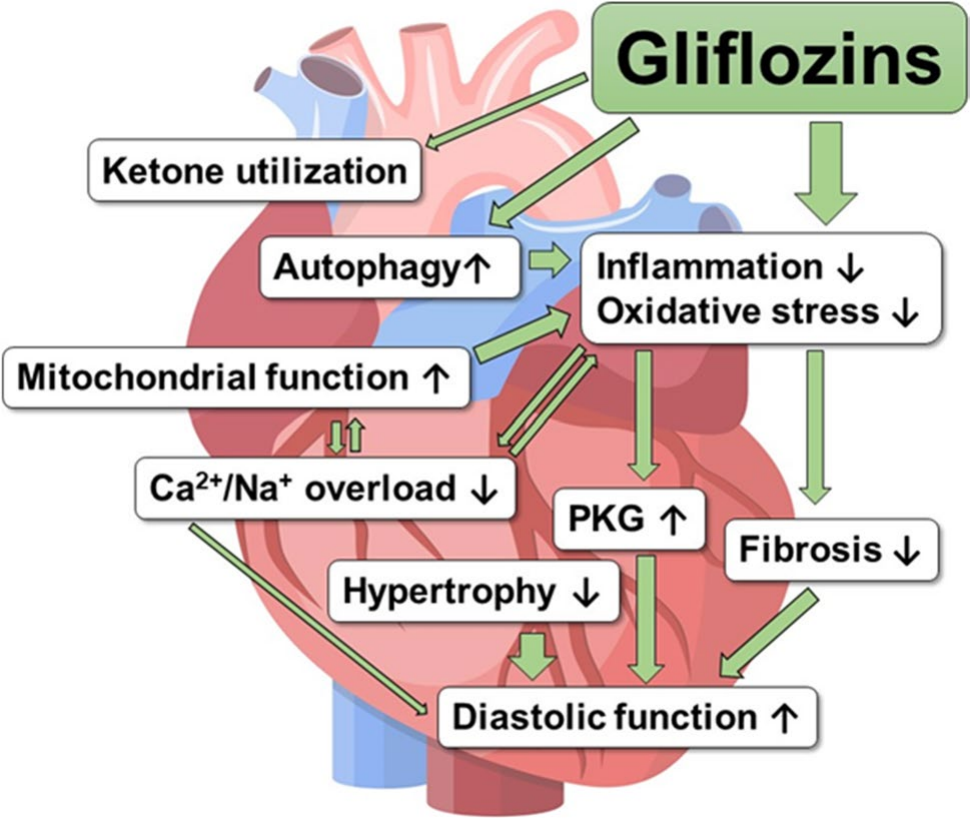
Proposed myocardial mechanisms of Gliflozins (SGLT2 inhibitors) in HF. PKG, Proteinkinase G. Image heart: ©AdobeStock/Rogatnev

A plethora of multidirectional mechanisms of SGLT2i, which could underlie the favourable HF outcomes, has been proposed. While improvement of conventional secondary risk factors is unlikely to explain the marked benefit of these drugs on HF end points, several novel mechanisms of SGLT2i were reported, including differential volume regulation, cardiorenal mechanisms, metabolic effects, improved cardiac remodelling, direct effects on cardiac contractility and ion-homeostasis, reduction of inflammation and oxidative stress as well as an impact on autophagy and adipokines. Importantly, differences in the investigated species and disease, the treatment protocols and many other methodological varieties make it somewhat difficult to extrapolate central mechanisms of SGLT2i. So far, evidence rather points towards multifactorial effects associated with reduced systemic and myocardial inflammation and oxidative stress leading to improved cardiac function (Fig. 4). Nevertheless, while many theories about the favourable mechanisms of SGLT2i in HF have been postulated, further experimental research is clearly warranted. Translational studies i.e. utilising human samples could help to better elucidate the mechanisms of SGLT2i in HF. In any case, SGLT2i may be better termed as gliflozins at least from a cardiovascular point of view, as they seem to have multiple additional effects.
